# A Support Vector Machine Based on Liquid Immune Profiling Predicts Major Pathological Response to Chemotherapy Plus Anti-PD-1/PD-L1 as a Neoadjuvant Treatment for Patients With Resectable Non-Small Cell Lung Cancer

**DOI:** 10.3389/fimmu.2021.778276

**Published:** 2021-12-15

**Authors:** Jie Peng, Dan Zou, Lijie Han, Zuomin Yin, Xiao Hu

**Affiliations:** ^1^ Department of Oncology, The Second Affiliated Hospital, Guizhou Medical University, Kaili, China; ^2^ Department of Radiation Oncology, Cancer Hospital of the University of Chinese Academy of Sciences, Hanzhou, China; ^3^ Department of Hematology, The First Affiliated Hospital of Zhengzhou University, Zhengzhou, China

**Keywords:** non-small cell lung cancer, support vector machine, major pathological response, liquid immune profiling, neoadjuvant treatment

## Abstract

The biomarkers for the pathological response of neoadjuvant chemotherapy plus anti-programmed cell death protein-1/programmed cell death-ligand 1 (PD-1/PD-L1) (CAPD) are unclear in non-small cell lung cancer (NSCLC). Two hundred and eleven patients with stage Ib-IIIa NSCLC undergoing CAPD prior to surgical resection were enrolled, and 11 immune cell subsets in peripheral blood were prospectively analyzed using multicolor flow cytometry. Immune cell subtypes were selected by recursive feature elimination and least absolute shrinkage and selection operator methods. The support vector machine (SVM) was used to build a model. Multivariate analysis for major pathological response (MPR) was also performed. Finally, five immune cell subtypes were identified and an SVM based on liquid immune profiling (LIP-SVM) was developed. The LIP-SVM model achieved high accuracies in discovery and validation sets (AUC = 0.886, 95% CI: 0.823–0.949, *P* < 0.001; AUC = 0.874, 95% CI: 0.791–0.958, *P* < 0.001, respectively). Multivariate analysis revealed that age, radiological response, and LIP-SVM were independent factors for MPR in the two sets (each *P* < 0.05). The integration of LIP-SVM, clinical factors, and radiological response showed significantly high accuracies for predicting MPR in discovery and validation sets (AUC = 0.951, 95% CI: 0.916–0.986, *P* < 0.001; AUC = 0.943, 95% CI: 0.912–0.993, *P* < 0.001, respectively). Based on immune cell profiling of peripheral blood, our study developed a predictive model for the MPR of patients with NSCLC undergoing CAPD treatment that can potentially guide clinical therapy.

## Introduction

The combination of anti-programmed death receptor 1 (PD-1), or its ligand (PD-L1), and chemotherapy has recently gained attention in patients with advanced non-small cell lung cancer (NSCLC). In the KEYNOTE-407, KEYNOTE-189, and IMPOWER-130 studies (1–3), platinum-based double-chemotherapy plus anti-PD-1/PD-L1 (such as atezolizumab and pembrolizumab) showed significantly higher objective response, longer progression-free survival (PFS), and overall survival (OS) than chemotherapy alone in patients with metastatic NSCLC. The latest CHECKMATE-816 study showed that neoadjuvant nivolumab plus chemotherapy significantly improves the major pathological response (MPR), as reported in the American Society of Clinical Oncology 2021 (abstract number: 8503). The tumor mutation burden and PD-L1 expression were not found to be related to MPR in patients with NSCLC treated with neoadjuvant chemotherapy plus anti-PD-1/PD-L1 (CAPD). A recent study (SAKK 16/14) reported that patients who achieved an MPR showed longer event-free survival and OS than patients who did not among patients who underwent a combination of perioperative durvalumab and neoadjuvant chemotherapy (4). However, only a subset of patients with NSCLC acquired an MPR when undergoing CAPD as a neoadjuvant treatment. Therefore, identifying novel and useful biomarkers to predict the patients most likely to acquire MPR from CAPD before surgery is critical.

Tumor-infiltrating lymphocytes and peripheral blood immune cells were found to be related to the response of solid tumors to therapy (5–9). Circulating exhausted-phenotype CD8^+^ T cells are associated with poor immunological response to pembrolizumab in patients with stage IV melanoma (10). Furthermore, circulating PD-1^+^CD8^+^ T cells, memory T cells, and elevated monocyte levels are strong predictors of response to immunotherapy (11–13). Therefore, we speculated that the immune cell subsets in peripheral blood may be associated with the MPR to CAPD as a neoadjuvant treatment of patients with NSCLC; if confirmed, an immune cell model can be built.

Machine learning has been gaining attention with respect to medical image recognition tasks and building prognostic prediction models from high-dimensional gene expression profiling data (14–18). A CHECKMATE-025 study developed a Bayesian network model for predicting immunotherapy prognosis in patients with metastatic renal cell carcinoma (19). Using radiomics biomarkers, radiology text reports, or somatic mutations, machine learning models could estimate the response and prognosis in patients with NSCLC treated with immunotherapy (20–22). To the best of our knowledge, the use of a support vector machine (SVM) based on immune cells to predict the MPR to CAPD treatment has never been reported.

Here, immune cell profiling was performed before the initial CAPD neoadjuvant therapy of patients with NSCLC before surgery. Using machine learning, a predictive immunological model was constructed and validated that can help identify patients who would most likely acquire MPR while undergoing CAPD.

## Methods

### Patients

Each patient provided detailed informed consent to the investigator. PD-L1 expression and EGFR/ALK mutation status were not necessary conditions for enrollment. The key eligibility criteria were as follows: (i) patients were at least 18 years old and willing to provide routine peripheral blood (2 mL) for immune cell analysis; (ii) Eastern Cooperative Oncology Group performance status was 0–1; (iii) a tissue biopsy of the tumor was confirmed to be lung adenocarcinoma (LUAD) or lung squamous cell carcinoma (LUSC) before any treatment; and (iv) patients with resectable stage Ib-IIIa NSCLC were examined using whole-body computed tomography (CT) or positron emission tomography-CT. The exclusion criteria were: (i) patients who could not tolerate treatment, such as those allergic to albumin-bound paclitaxel; (ii) patients undergoing CAPD but whose NSCLC was progressing rapidly or showed organ metastasis and were not suitable for resection treatment. Between September 2019 and June 2021, 211 patients who were receiving neoadjuvant treatment before surgery and met the criteria were recruited at the Cancer Hospital of the University of Chinese Academy of Sciences (CHUCAS) (**Supplementary Figure 1**). The patients were randomly divided in a 3:2 ratio into a discovery set (n = 127) and validation set (n = 84). The institutional review board of the Second Affiliated Hospital of Guizhou Medical University and CHUCAS approved our clinical research design. We have been conducted in accordance with the World Medical Association’s Declaration of Helsinki.

### Study Design

**Figure 1:**
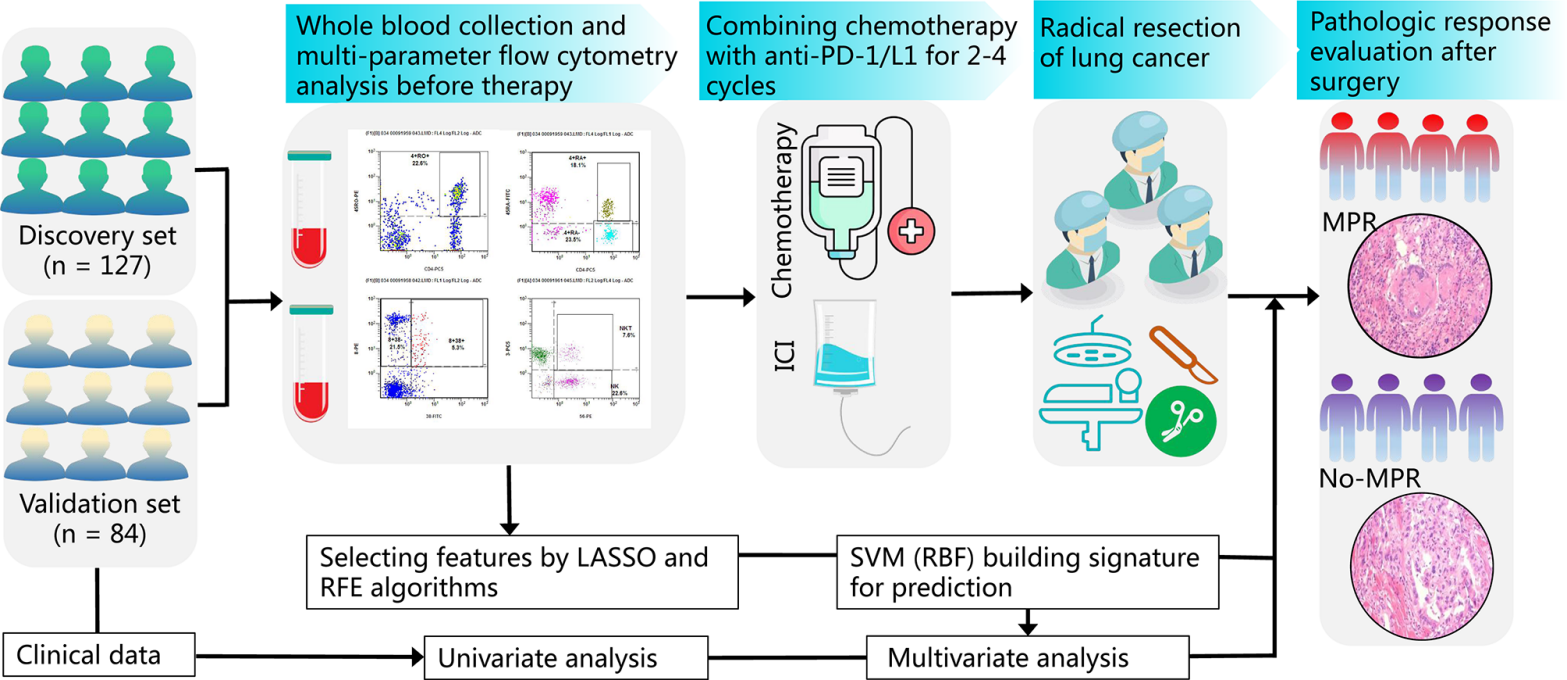
The study flowchart from patient enrollment to machine learning. Whole blood samples from patients with NSCLC on an empty stomach were collected and analyzed by multicolor flow cytometry before treatment. Patients with NSCLC were subjected to neoadjuvant CAPD treatment followed by radical surgery of lung cancer and MPR evaluation of tumor tissues. The association between clinical factors, radiological response, and MPR was analyzed by univariate analysis. The immune cells were selected by RFE and LASSO methods. The LIP-SVM signature was built and tested in the discovery and validation sets. Then, multivariate analysis for MPR was performed by logistic regression. Finally, three models integrating LIP-SVM, clinical factors, or radiological response were evaluated for predicting MPR. NSCLC, non-small cell lung cancer; CAPD, chemotherapy plus anti-PD-1/PD-L1; MPR, major pathological response; RFE, recursive feature elimination; LASSO, least absolute shrinkage and selection operator; LIP-SVM, support vector machine model based on liquid immune profiling.

A flowchart of the study design is shown in **Figure 1**. Before treatment, whole blood samples were collected from patients and rapidly tested by multicolor flow cytometry before initial treatment. The detailed results of peripheral immune cells and clinical characteristics were recorded. Two hundred and eleven patients with NSCLC received 2–4 cycles of CAPD. According to the MPR status, the doctors then performed radical surgery of lung cancer. The pathological response of tumor tissues was estimated by a senior pathologist. We then analyzed the association between clinical factors and MPR using univariate analysis of both cohorts (discovery and validation sets). The immune cells in peripheral blood were chosen by recursive feature elimination (RFE) and least absolute shrinkage and selection operator (LASSO) methods. After integrating these two selection methods, the final immune cell subtypes were confirmed. An SVM model based on liquid immune profiling (LIP-SVM) was developed and validated in the two cohorts. Multivariate analysis for MPR was performed for patients with NSCLC using logistic regression. The integration of LIP-SVM, clinical factors, or radiological response was evaluated in terms of predictive accuracy for MPR. Contrast-enhanced CT images were examined to clearly identify the primary tumor. The radiological response, including complete response (CR), partial response (PR), stable disease (SD), and progressive disease (PD), was estimated before radical surgery by a senior thoracic radiologist using Response Evaluation Criteria in Solid Tumors (RECIST) version 1.1.

### Assessment of MPR for Patients Receiving CAPD

MPR is defined as the reduction of viable tumors to clinically defined significant margins, depending on the particular histological type of lung cancer and the specific treatment type. All histological types of lung cancer had an MPR with a histological definition of less than or equal to 10% of the viable tumor. The MPR was calculated by dividing the size of the viable tumor by the size of the tumor bed. Here, this was used to establish the threshold of the number of clinical trials. The pathology report recorded the total number of masses in the tumor bed, including some uninvolved lungs, even if these masses were not entirely composed of the tumor bed. The MPR can also be classified as a primary pulmonary tumor, where little or no viable metastatic carcinomas were found in the lymph nodes (ypT0, N1, 2, 3).

### Neoadjuvant Treatment Strategy of CAPD

Patients with LUAD preoperatively received a folic acid, vitamin B12, and glucocorticoid pretreatment that is prescribed according to the local guidelines for pemetrexed. All patients received intravenous injections of cisplatin (75 mg/m^2^, d1) or carboplatin (under the concentration-time curve, 5 mg/mL/min, d1) and pemetrexed (500 mg/m^2^, d1) for 2–4 cycles. Patients with LUSC preoperatively received intravenous injections of cisplatin (75 mg/m^2^, d1) or carboplatin (under the concentration-time curve, 5 mg/mL/min, d1) and nab-paclitaxel (135 mg/m^2^, d1, d8) for 2–4 cycles. The anti-PD-1 regimen included camrelizumab (3 mg/kg, Q2W), pemetrexed (500 mg/m^2^, Q3W), nivolumab (3 mg/kg, Q2W), toripalimab (3 mg/kg, Q2W), tislelizumab (200 mg/m^2^, Q3W), or sintilimab (200 mg, Q3W), whereas the anti-PD-L1 regimen included durvalumab (10 mg/kg, Q2W); the patients received both injections following each chemotherapy cycle. Chemotherapy and anti-PD-1/PD-L1 regimens were administered every 3 weeks to patients with LUAD and LUSC.

### Defining and Profiling the Immune Cell Subtypes in Peripheral Blood

We evaluated four types of circulating immune cells: B, T, natural killer (NK), and natural killer T (NKT) cells. B and T cells were defined by CD19 expression (CD19^+^ B cells) and CD3 expression (CD3^+^ T cells), respectively. The presence of CD8 and CD4 was used to identify T-lymphocyte subsets (CD3^+^CD8^+^ T cells and CD3^+^CD4^+^ T cells). Memory (CD4^+^CD45RO^+^) T cells and CD4^+^ naïve (CD4^+^CD45RA^+^) T cells were identified by CD45RA and CD45RO expression. A combination of CD56 and CD3 was used to identify NKT (CD3^+^CD56^+^) and NK (CD3^-^CD56^+^) lymphocyte subsets. Activated CD8^+^ T cells (CD8^+^CD38^+^ T cells) were recognized by CD38 expression. BD Biosciences (San Jose, CA, USA) provided the following antibodies in **Supplementary Table 1**: CD8-FITC (#555366), CD4-FITC (#550628), CD3-FITC (#555332), CD56-FITC (#55664), CD19-FITC (#555412), CD45RO-APC (#559865), CD38-PE (#555460), CD45RA-PE (#555489), and FITC/APC/PE controls (#55749; #555748; #5555776). For lymphocyte staining, 4 mL of peripheral blood was collected into a blood tube with ethylenediaminetetraacetic acid and an anticoagulant. Next, 20 μL of CD3-FITC/CD56-PE, CD19-FITC, CD4-FITC/CD45RO-APC/CD45RA-PE, CD8-FITC/CD38-PE, and FITC/PE/APC isotype controls was separately added to five flow cytometry tubes and 100 μL of every blood sample was added to every test tube. The tubes were sufficiently mixed with corresponding antibodies in the dark and incubated for 30 min at room temperature (20°C). A hemolytic agent (up to 2 mL; #70-LSB3; BD Biosciences) was then added to every tube. The supernatant was then removed by centrifugation (6 min), washed twice with phosphate-buffered saline (#SH300256; Hyclone, Logan, UT, USA), and resuspended in paraformaldehyde. Flow cytometry (FACSVia; BD Biosciences) was used to examine the cells. More than 2,000 cells were detected at the lymphocyte gate in every sample. CellQuest Pro software (BD Biosciences) was used to analyze the percentages of positively labeled lymphocytes. The staining procedure was completed and analyzed within 24 h after blood collection.

### Feature Selection of RFE and LASSO Algorithms

Two feature selection methods (RFE and LASSO) were used in this study. RFE recursively reduces the size of the examined feature set to select features. The prediction model based on the original features is trained and a weight is assigned to each feature. Features with a minimum absolute weight are recursively removed until the desired number is reached. Random forest function and 5-fold cross-validation (CV) sampling were used for RFE. In addition, we used LASSO to select the most important immune cells from the discovery set. A log partial likelihood subject based on the LASSO method is minimized to add the absolute values of the parameters. Here, the standardized constraint parameter was set to -1.434. RFE and LASSO were performed using the “caret” package in R version 3.5.1.

### SVM Building Model

The SVM is a classical model of machine learning with important value in tumor classification, prognosis, and treatment response predictions (23). Radial basis function, the most popular kernel function of SVM for nonlinear classification, can significantly improve the classification ability of the SVM by mapping the original input space to the feature space. The original nonlinear input space is transformed into the linear separability space and classified linearly in the feature space. The equation we used was as follows:


$$K(x,z)=\exp(-\gamma {\left|x-z\right|}^{2})$$

where γ is greater than 0 and the parameters need to be adjusted. The tuning parameters were set as sigma = 0.035, C = 100, and cross = 10. The “kernlab” library was implemented using R software.

### Statistical Analysis

R software and GraphPad Prism were used to perform statistical analysis. A correlation heatmap was generated using the “pheatmap” package to depict the relationships between immune cells in the discovery and validation sets. Correlation scatter plots were used to indicate associations between immune cells. The scatter dot and box plots were used to represent the median and 95% confidence intervals (CI). The differences between groups were analyzed using the Mann–Whitney U test. The receiver operating characteristic curves (ROC) were plotted using the “pROC” package. The frequencies of two groups were compared using the chi-square test. Univariate and multivariate logistic analyses for MPR were performed in the two sets. Statistical significance was defined as *P*-value < 0.05.

## Results

### Clinical Characteristics and MPR

The baseline characteristics of 211 patients from two independent cohorts are presented in **Table 1**. Most patients (92.42%) were male, old (> 60 years; 60.67%), smokers (78.67%), and with squamous cancer (82.46%). Stage IIIa was assigned to 68.72% of patients, 89.57% received anti-PD-1 immunotherapy, 70.61% underwent two treatment cycles, and 45.50% of cases experienced radiological PR (**Table 1**). The pathological response was MPR or no-MPR in the discovery (46.46% or 36.90%) and validation (53.54% or 63.10%) sets, respectively. No variables significantly differed between the two cohorts (*P* > 0.05).

**Table 1:** Characteristics of patients in the discovery and validation sets.

Variable	Patients N = 211 (%)	Discovery set (n = 127)	Validation set (n = 84)	*P*-value^a^
Gender				0.737
Female	16 (7.58%)	9 (7.08%)	7 (8.33%)	
Male	195 (92.42%)	118 (92.92%)	77 (91.67%)	
Age (years)				0.556
≤ 60	83 (39.33%)	52 (40.94%)	31 (36.90%)	
> 60	128 (60.67%)	75 (59.06%)	53 (63.10%)	
Smoking status				0.976
Smoker	45 (21.33%)	27 (21.26%)	18 (21.43%)	
Non-smoker	166 (78.67%)	100 (78.74%)	66 (78.57%)	
Histology				0.787
Squamous	174 (82.46%)	104 (81.89%)	70 (83.33%)	
Adenocarcinoma	37 (17.54%)	23 (18.11%)	14 (16.67%)	
Stage				0.122
Ib-IIb	67 (31.68%)	35 (27.56%)	32 (36.90%)	
IIIa	145 (68.72%)	92 (72.44%)	53 (63.10%)	
Immunotherapy				0.911
Anti-PD-1	189 (89.57%)	114 (89.76%)	75 (89.29%)	
Anti-PD-L1	22 (10.42%)	13 (10.24%)	9 (10.71%)	
Cycles				0.183
2	149 (70.61%)	94 (74.01%)	55 (65.47%)	
3-4	62 (29.39%)	33 (25.99%)	29 (34.53%)	
Radiological response				0.225
CR	48 (22.75%)	34 (26.77%)	14 (16.67%)	
PR	96 (45.50%)	52 (42.52%)	44 (52.38%)	
SD	57 (27.01%)	36 (28.35%)	21 (25.00%)	
PD	10 (4.74%)	5 (2.36%)	5 (5.95%)	
Pathologic response				0.170
MPR	90 (42.65%)	59 (46.46%)	31 (36.90%)	
No MPR	121 (57.35%)	68 (53.54%)	53 (63.10%)	

a P-value is derived from the difference in clinical characteristics between the discovery data set and the validation data set.

CR, complete response; PR, partial response; SD, stable disease; PD, progressive disease; MPR, major pathological response.

By analyzing the association between clinical data and MPR in the discovery and validation sets, we found that old age (> 60 y) was significantly correlated with MPR (*P* = 0.023) (**Supplementary Figure 2A**), patients with squamous cancer presented with a higher MPR than patients with adenocarcinoma (*P* = 0.025) (**Supplementary Figure 2B**), and patients who underwent an anti-PD-L1 regimen showed a higher MPR than those who underwent an anti-PD-1 regimen (*P* = 0.023) (**Supplementary Figure 2C**). Moreover, patients with radiological CR and PR showed a higher MPR than patients with radiological SD or PD (both *P* < 0.001) (**Supplementary Figure 2D**). The detailed results of the two cohorts are presented in **Supplementary Table 2**.

### Most Immune Cells Are Similar Between the Discovery and Validation Sets

**Figure 2:**
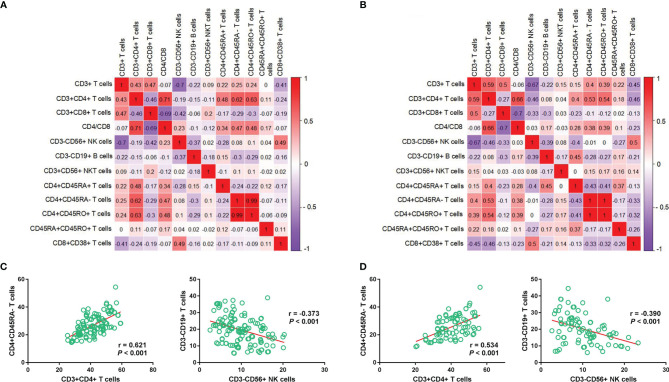
Correlation between immune cell subtypes in the discovery and validation sets. Correlation heatmap depicts the relationship between each immune cell subtype in the discovery **(A)** and validation sets **(B)**, respectively; Relationship between CD4^+^CD45RA^-^ T cells and CD3^+^CD4^+^ T cells/CD4^+^CD45RA^-^ T cells and CD3^+^CD4^+^ T cells in the discovery **(C)** and validation **(D)** cohorts.

The results of the discovery set were similar with those of the validation set (**Figures 2A, B**). The relative abundance of CD4^+^CD45RA^-^ and CD3^+^CD4^+^ T cells showed a positive correlation (r = 0.621, *P* < 0.001; r = 0.534, *P* < 0.001), whereas that of CD3^-^CD19^+^ B cells and CD3^-^CD56^+^ NK cells showed a negative correlation (r = -0.373, *P* < 0.001; r = -0.390, *P* < 0.001) (**Figures 2C, D** **)**.

### Five Immune Cells Were Selected and Significantly Associated With MPR

**Figure 3:**
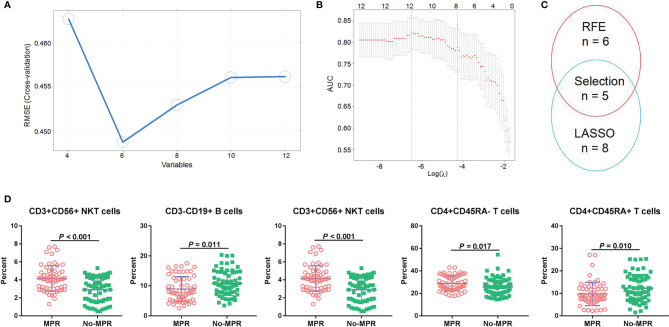
Immune cell selection and correlation with MPR. RFE **(A)** and LASSO **(B)** algorithms were used to perform optimal feature selection; Overlapping features were selected using two algorithms **(C)**; Percentages of immune cells were compared between the MPR and no-MPR groups in patients with NSCLC from the discovery and validation sets treated with CAPD **(D)**. MPR, major pathological response; RFE, recursive feature elimination; LASSO, least absolute shrinkage and selection operator; NSCLC, non-small cell lung cancer; CAPD, chemotherapy plus anti-PD-1/PD-L1.

To find suitable predictors for MPR, the immune cells before neoadjuvant therapy were identified, and then RFE and LASSO were used to perform feature selection. Based on 5-fold CV analysis, root mean square error (RMSE) showed that a combination of six variables had the smallest errors, and thus these immune cells were selected **(** **Figure 3A** **)**. Then, LASSO coefficient analysis of 12 features of immune cells was performed, after which eight coefficients were chosen based on a 5-fold CV analysis of minimum criteria **(** **Figure 3B** **)**. To find robust features, we selected five immune cell types with overlapping features for the SVM model **(** **Figure 3C** **)**. CD3^+^CD56^+^ NKT cells were found significantly associated with MPR, and can thus be a predictor for MPR (*P* < 0.001, **Figure 3D**). In the discovery set, the percentage of CD3^-^CD19^+^ B cells was higher in the no-MPR group (*P* = 0.011, **Figure 3D**), whereas that of CD3^-^CD56^+^ NK cells was higher in the MPR group (*P* = 0.032). Moreover, patients in the MPR group had a higher percentage of CD4^+^CD45RA^-^ T cells than those in the no-MPR group (*P* = 0.017). The percentage of CD4^+^CD45RA^+^ T cells was higher in the no-MPR group than in the MPR group.

### LIP-SVM Was Built and Validated in Another Independent Cohort

**Figure 4:**
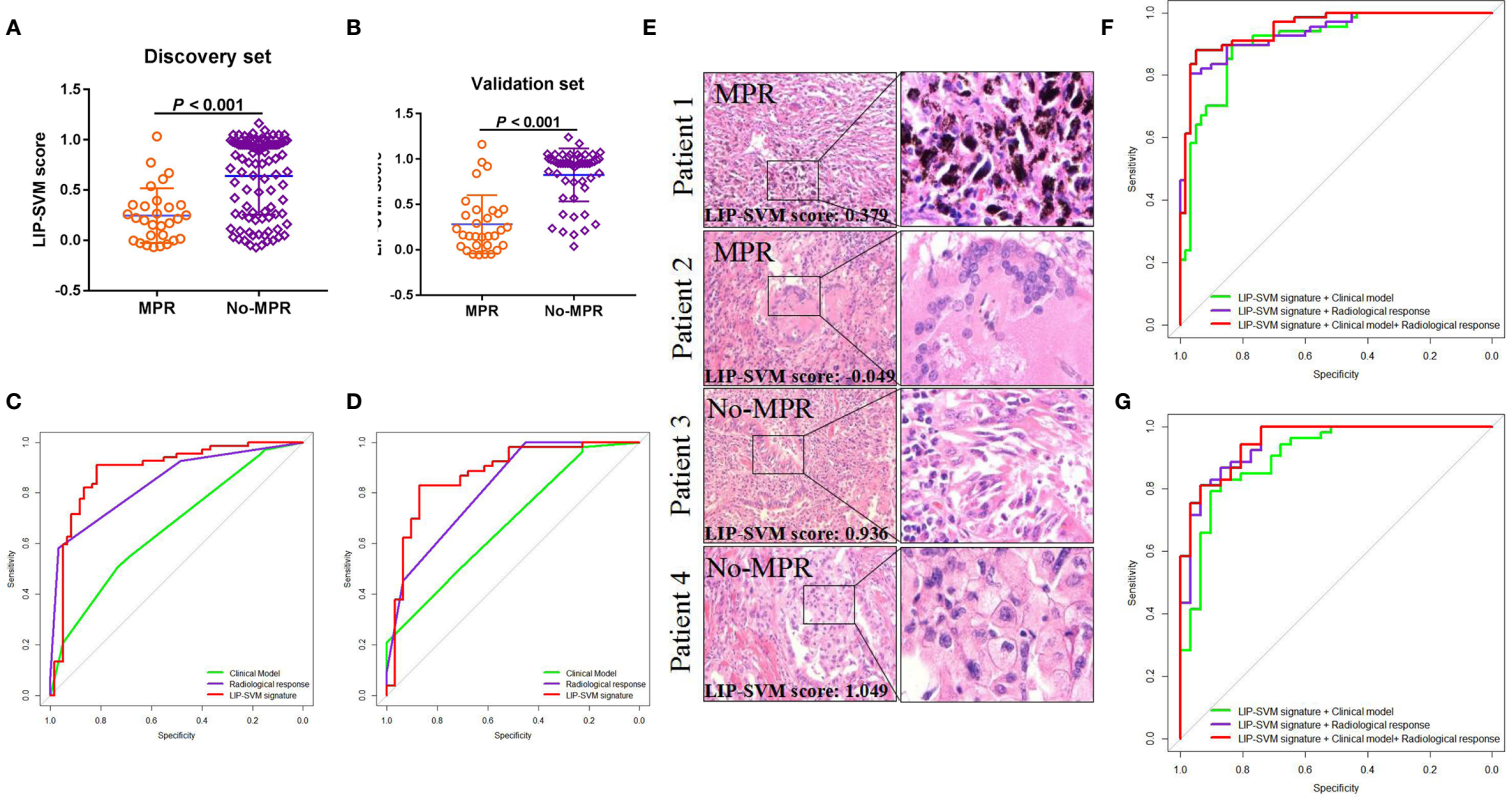
LIP-SVM score was compared, and signatures were predicted for MPR using the two sets. LIP-SVM score comparisons between MPR and no-MPR groups of patients with NSCLC in the discovery **(A)** and validation sets **(B)**. The area under the ROC curves of LIP-SVM signature, clinical model, and radiological response were plotted and compared for the discovery **(C)** and validation **(D)** sets; LIP-SVM score for each patient with NSCLC treated with CAPD **(E)**; The clinical model included age, histology, and immunotherapy. The area under the ROC curves of the LIP-SVMRC model, LIP-SVM signature combined with the clinical model, and LIP-SVM signature combined with radiological response were plotted for the discovery **(F)** and validation **(G)** sets. LIP-SVM, support vector machine model based on immune cell profiling; MPR, major pathological response. LIP-SVM, support vector machine model based on immune cell profiling; ROC, receiver operating characteristic; NSCLC, non-small cell lung cancer; CAPD, chemotherapy plus anti-PD-1/PD-L1; LIP-SVMRC, LIP-SVM signature plus radiological response plus clinical model.

To develop the SVM model, fine-tuning was performed during the training process. LIP-SVM was calculated as the output score of machine learning in the discovery and validation sets. We found that patients in the MPR group showed significantly higher LIP-SVM scores than patients in the no-MPR group in both cohorts (both *P* < 0.001; **Figures 4A, B** **)**. To compare the accuracy of different models or predictors in our study, we first used three meaningful clinical features, including age (≤ 60 vs. > 60 years), histology (squamous vs. adenocarcinoma), and immunotherapy (anti-PD-1 vs. anti-PD-L1), to build a clinical model using logistic regression [area under the curve (AUC) = 0.663, 95% CI: 0.568–0.756, *P* = 0.001; AUC = 0.680, 95% CI: 0.565–0.796, *P* = 0.005, respectively; **Figures 4C, D**]. Moreover, the radiological response (CR, PR, SD, and PD) was also a strong indicator for predicting MPR of the two sets (AUC = 0.840, 95% CI: 0.772–0.908, *P <* 0.001; AUC = 0.820, 95% CI: 0.727–0.913, *P <* 0.001, respectively; **Figures 4C, D** **)**. Comparing the above two predictors, the LIP-SVM signature showed the higher accuracy in the discovery and validation sets (AUC = 0.886, 95% CI: 0.823–0.949, *P <* 0.001; AUC = 0.874, 95% CI: 0.791–0.958, *P <* 0.001, respectively; **Figures 4C, D** **)**. To better understand the utility of LIP-SVM, pathological images from four patients in validation cohorts are presented. After 2–4 cycles of neoadjuvant CAPD before resection of lung cancer, patients 1 and 2 with MPR had lower LIP-SVM scores than patients 3 and 4 with no-MPR (0.379 and -0.049 vs. 0.936 and 1.049; **Figure 4E** **)**. The model is now available for free online testing (https://pengjie.shinyapps.io/lipsvm/).

### LIP-SVM Was an Independent Indicator of MPR in Patients With NSCLC

Based on the multivariate logistic regression analysis in the discovery set, age, radiological response, and LIP-SVM signature were independent risk factors for MPR [*P* < 0.016, *P* < 0.001, and *P* < 0.001; OR = 0.169 (0.034–0.649), 7.318 (3.147–20.588), and 29.788 (1.572–56.574), respectively; **Table 2**]. Age, radiological response, and LIP-SVM signature were also independent risk factors for MPR in the validation set [*P* = 0.046, *P* = 0.002, and *P* < 0.001; OR = 0.162 (0.019–0.990), 15.352 (3.487–27.805), and 28.186 (1.096–58.460), respectively]. To further improve the predictive accuracy, we integrated a combination of clinical factors or radiological response for multivariate analysis; we found that the LIP-SVMRC (LIP-SVM signature + radiological response + clinical model) model had the highest accuracy in the discovery and validation sets (AUC = 0.951, 95% CI: 0.916–0.986, *P <* 0.001; AUC = 0.943, 95% CI: 0.912–0.993, *P <* 0.001, respectively; **Figures 4F, G** **)**. Two models (LIP-SVM signature + radiological response and LIP-SVM signature + clinical model) also exhibited high predictive accuracy for MPR in the discovery (AUC = 0.935, 95% CI: 0.895–0.975, *P <* 0.001; AUC = 0.907, 95% CI: 0.855–0.959, *P <* 0.001, respectively) and validation (AUC = 0.931, 95% CI: 0.899–0.994, *P <* 0.001; AUC = 0.905, 95% CI: 0.837–0.934, *P <* 0.001, respectively; **Figures 4F, G** **)** sets.

**Table 2:** Multivariate analysis for MPR in the discovery and validation sets.

Variable	Discovery set (n = 127)		Validation set (n = 84)	
	OR (95% CI)	*P-*value	OR (95% CI)	*P-*value
Age (years) (≤ 60 vs. > 60)	0.169 (0.034–0.649)	0.016*	0.162 (0.019–0.990)	0.046*
Histology (squamous vs. adenocarcinoma)	1.698 (0.274–11.120)	0.569	16.908 (0.576–18.570)	0.195
Immunotherapy (anti-PD-1 vs. anti-PD-L1)	0.179 (0.013–1.872)	0.168	0.466 (0.013–8.458)	0.629
Radiological response (CR/PR vs. SD/PD)	7.318 (3.147–20.588)	< 0.001*	15.352 (3.487–27.805)	0.002*
LIP-SVM signature (Low score vs. High score)	29.788 (1.572–56.574)	< 0.001*	28.186 (1.096–58.460)	< 0.001*

OR, odds ratio; CI, confidence interval; LIP-SVM, support vector machines based on liquid immune profile; CR, complete response; PR, partial response; SD, stable disease; PD, progressive disease.

*P-value < 0.05.

## Discussion

In this prospective study, we selected five immune cell subtypes in peripheral blood and found that patients with three favorable immune cells (CD3^+^CD56^+^ NKT, CD3^-^CD56^+^ NK, and CD4^+^CD45RA^-^ T cells) had a high MPR. Based on the SVM algorithm, the LIP-SVM signature was developed and can be used to predict the MPR of CAPD treatment. Multivariate analysis for MPR in the discovery and validation sets revealed that old age, radiological response, and LIP-SVM signature were positive independent factors of MPR. Combined with clinical factors, radiological response, and LIP-SVM, the LIP-SVMRC model exhibited the highest accuracy for predicting MPR compared with the other two models. These findings indicate that LIP-SVMRC can be used as a novel tool for the effective identification of patients that may acquire MPR from CAPD.

Several studies have reported that the combination of chemotherapy and anti-PD-1/PD-L1 treatment for metastatic NSCLC as a first-line therapy significantly improves the treatment response, OS, and PFS of patients (24–27). However, some studies have reported that neoadjuvant combination immunotherapy, especially CAPD (4, 28), makes it challenging to identify significant biomarkers for predicting MPR in patients with NSCLC undergoing CAPD. In our study, we found that old (> 60 years) patients with squamous cancer had higher positive CAPD response, indicating that the combination of platinum-based double-chemotherapy (nab-paclitaxel/pemetrexed) and immunotherapy was more suitable for these patients. Although the sample size of patients on anti-PD-L1 was small (13 and 9 patients in the discovery and validation sets, respectively), the combination anti-PD-L1 regimen showed a higher MPR than the combination anti-PD-1 as a neoadjuvant treatment for patients with NSCLC. This may be because anti-PD-L1 treatment affects both the tumor microenvironment [e.g., T and B cells, dendritic cells (DCs), and macrophages] and the tumor itself, which frequently express PD-L1 (29, 30). In the evaluation of treatment response, we found that there was a partial discrepancy between radiological and pathological methods; nevertheless, radiological evaluation of neoadjuvant treatment response can aid preoperative prediction of MPR.

In addition to clinical factors and radiological response, detailed knowledge of the patient’s immune status of peripheral blood is needed to evaluate the efficacy of combination anti-PD-1/PD-L1 treatment, and tumor immunogenicity score is evaluated as a predictor of immune checkpoint inhibitor response (31, 32). Previous studies have reported that specific PD-1^+^CD56^+^ and CD8^+^ T cells frequently indicate a good prognosis in patients with melanoma treated with immunotherapy (33, 34). However, the immunological biomarkers for predicting MPR with CAPD as a first-line neoadjuvant remain unclear. The main reason for this is the paucity of reported data on neoadjuvant immunotherapy. In our prospective study, we revealed five immune cell subtypes based on liquid immune profiling for predicting the MPR of patients with stage Ib-IIIa NSCLC treated with CAPD. NKT cells have been reported to play a critical role in inducing cross-talk of plasmacytoid DCs with conventional DCs, which is associated with the generation of memory CD8^+^ T cells (35). A recent study reported that elevated peripheral NK cell numbers in patients with NSCLC are associated with responses (CR/PR) to immunotherapy (36). Our study revealed the positive correlations between CD3^+^CD56^+^ NKT or CD3^-^CD56^+^ NK cells and the MPR to CAPD. NKT/NK cells may play important roles in antitumor immunity, such as reactivation of fatigued immune cells derived from the tumor microenvironment.

Although immunotherapy can unleash CD8^+^ T cells and specific mutation-associated neoantigens, some tumor microenvironment factors (such as hypoxia and toxic metabolites) inhibit T cell activation (37, 38). A recent study showed that PD-1^+^CD8^+^ T cell-positive tumors are significantly associated with poor response to anti-PD-1 therapy (39). An increase in circulating PD-1^+^CD8^+^ T cell numbers in the early stage of immunotherapy is also an indicator of poor prognosis in advanced cancers (5). In our study, we found that active CD8^+^CD38^+^ T cells of the peripheral blood were not associated with MPR in patients undergoing neoadjuvant CAPD, indicating that a subtype of CD8^+^ T cells was suppressed or exhausted. In a recent study, the change of abundance in CD4^+^CD45RA^+^ T cells was a predictive biomarker for PFS after chemoradiotherapy (40). Interestingly, CD4^+^CD45RA^-^ T cells were positively and CD4^+^CD45RA^+^ T cells were negatively correlated with MPR in our study. These results suggest that a subtype of CD4^+^ T cells plays a crucial role in determining immunotherapy response in the initial stage of CAPD treatment. Moreover, circulating CD3^-^CD19^+^ B cell counts are increased in patients with oral squamous cell carcinoma after radical operation or chemotherapy (41), but their value in predicting treatment response and prognosis is unclear. In our study, we found that patients with neoadjuvant CAPD and acquired MPR exhibited a lower percentage of CD3^-^CD19^+^ B cells than patients without MPR. This is the first report of an association between CD3^-^CD19^+^ B cells and CAPD, revealing their potentially negative role in cancer treatment response or prognosis.

According to RECIST V.1.1, most patients (68.25%) acquired an objective response (CR/PR) to combination treatment, but this radiological method had a high specificity and low sensitivity in the two sets (specificity = 96.67 and 93.55; sensitivity = 58.21 and 45.28, respectively), which is not accurate enough to preoperatively predict pathological response. To precisely screen the patients for MPR, a machine learning method (integrating RFE, LASSO, and SVM algorithms) based on immune cell profiling was performed in this study. After fine-tuning the parameters, the LIP-SVM model exhibited higher predictive accuracy than the clinical model and evaluation of radiological response. Moreover, the LIP-SVM model showed an earlier prediction of MPR before initial CAPD than radiological estimation. Multivariate analysis for MPR also revealed that the LIP-SVM signature was an independent factor in both cohorts. Several studies have integrated clinical factors and series models to improve the accuracy and robustness capability (42–44). In our study, the AUC of the LIP-SVMRC model, which integrates three factors (immune cells, radiological evaluation, and clinical variables), was high in all models, indicating a greatly improved predictive accuracy. This preoperative prediction model of MPR may be helpful for guiding radical surgery and personalizing a treatment regimen for each patient.

Our study had two limitations. First, because of the high cost of analysis, targeted next-generation sequencing or whole-exome sequencing results were not analyzed in all samples from patients with NSCLC, which led to a small percentage of patients with test results that could not be analyzed. Second, our sample size was not large and multi-center prospective cohorts are required.

In conclusion, our study revealed the significant association between clinical variables (old age, squamous cancer, and anti-PD-L1 treatment), radiological response, and immune cells from peripheral blood and MPR in patients with NSCLC receiving 2–4 cycles of neoadjuvant CAPD. The classifications of the SVM model based on immune cell profiling and integration models provide a novel and noninvasive predictive method for identifying patients who may achieve MPR after CAPD.

## Data Availability Statement

The raw data supporting the conclusions of this article will be made available by the authors, without undue reservation.

## Ethics Statement

The studies involving human participants were reviewed and approved by the Second Affiliated Hospital of Guizhou Medical University and the Cancer Hospital of the University of Chinese Academy of Sciences. The patients/participants provided their written informed consent to participate in this study.

## Author Contributions

Conception and design: JP. Administrative support: JP. Provision of study materials or patients: JP and DZ. Collection and assembly of data: JP. Data analysis and interpretation: JP. Manuscript writing: All authors. All authors contributed to the article and approved the submitted version.

## Funding

This work was supported by the Qian Dong Nan Science and Technology Program [qdnkhJz2020-013] and the Science and Technology Foundation of Guizhou Province [Grant No. Qian ke he ji chu-ZK 2021, yi ban 454], the Science and Technology Fund Project of Guizhou Provincial Health Commission [gzwjkj2019-1-077].

## Conflict of Interest

The authors declare that the research was conducted in the absence of any commercial or financial relationships that could be construed as a potential conflict of interest.

## Publisher’s Note

All claims expressed in this article are solely those of the authors and do not necessarily represent those of their affiliated organizations, or those of the publisher, the editors and the reviewers. Any product that may be evaluated in this article, or claim that may be made by its manufacturer, is not guaranteed or endorsed by the publisher.
