# Supplementary material for: A Support Vector Machine Based on Liquid Immune Profiling Predicts Major Pathological Response to Chemotherapy Plus Anti-PD-1/PD-L1 as a Neoadjuvant Treatment for Patients With Resectable Non-Small Cell Lung Cancer
Source: Front Immunol. 2021 Dec 15;12:778276. doi: 10.3389/fimmu.2021.778276 (PMC8797141; doi:10.3389/fimmu.2021.778276)
Supplement: Supplementary file 1 [file DataSheet_1.docx]

Supplementary Material

# Supplementary Figures and Tables

## Supplementary Figures


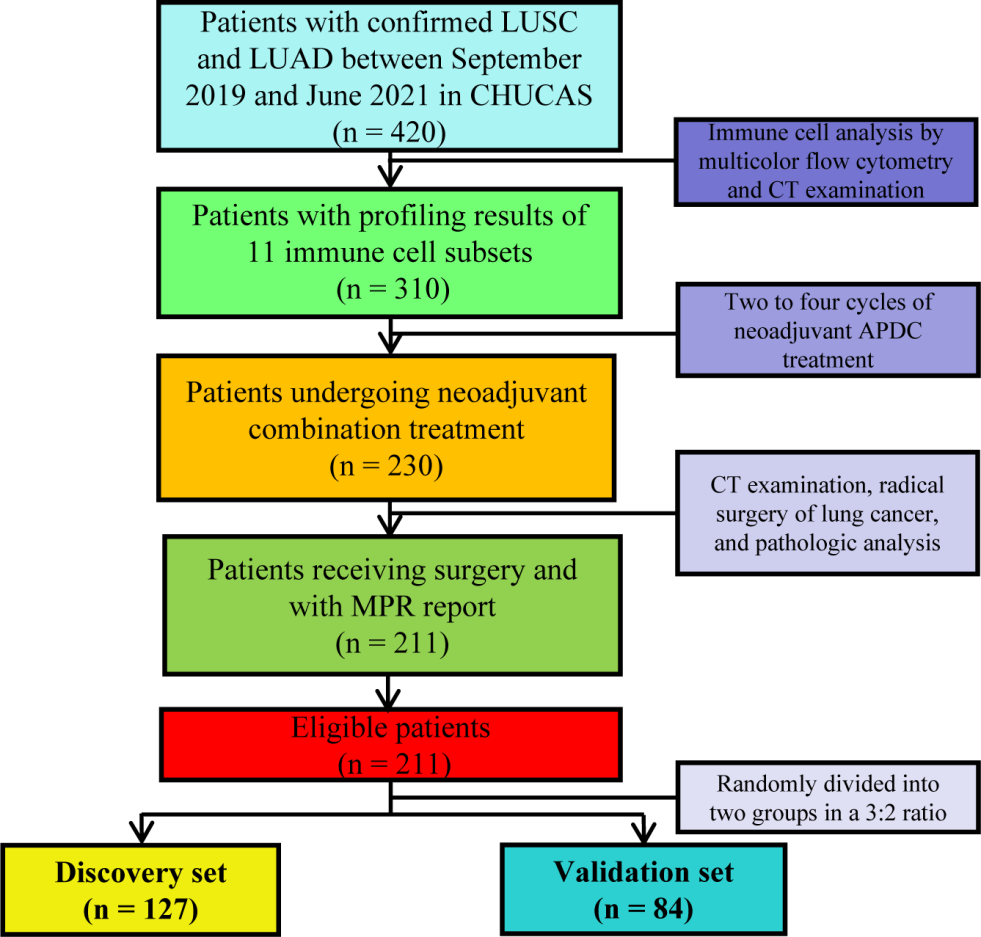


**Supplementary Figure 1.** Flowchart of patient enrollment at the Cancer Hospital of the University of Chinese Academy of Sciences (CHUCAS). Patients meeting the eligibility criteria were recruited. During recruitment, four patients were found to be allergic to albumin-bound paclitaxel during the first cycle of APDC treatment; APDC treatment was discontinued for these patients. Moreover, three patients were found to have distant metastasis by CT scan during APDC treatment. Abbreviations: LUSC, lung squamous cell carcinoma; LUAD, lung adenocarcinoma; MPR, major pathological response; CT, computed tomography; CAPD, chemotherapy plus PD-1/PD-L1.


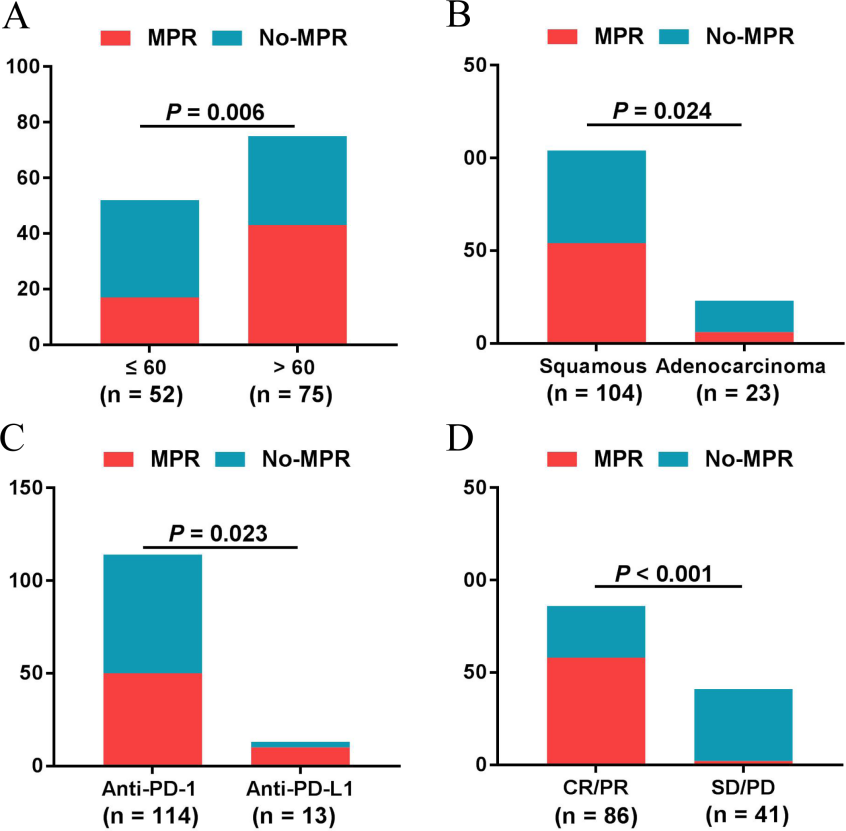


**Supplementary Figure 2.** Correlation between clinical factors, radiological response, and MPR. A–D, Relationship between age (A); squamous cancer vs. adenocarcinoma (B); anti-PD-L1 vs. anti-PD-1 immunotherapy; and radiological responses (CR/PR vs. SD/PD) (D) with MPR in the discovery and validation sets. Abbreviations: MPR, major pathological response; anti-PD-L1, programmed cell death-ligand-1; anti-PD-1, anti-programmed cell death protein-1; CR, complete response; PR, partial response; SD, stable disease; PD, progressive disease.

## Supplementary Tables

**Supplementary Table 1. Antibodies of immune cells profiling**

| **Company name** | **Antibody catalog number (n = 11)** |
| --- | --- |
| BD Biosciences | CD8-FITC (#555366)  CD4-FITC (#550628)  CD3-FITC (#555332)  CD56-FITC (#55664)  CD19-FITC (#555412)  CD45RO-APC (#559865)  CD38-PE (#555460)  CD45RA-PE (#555489)  FITC/APC/PE controls ( #55749; #555748; #5555776) |
|  | |

**Supplementary Table 2. Univariate analysis for MPR in the discovery and validation sets**

| Variable | Univariate analysis | |
| --- | --- | --- |
|  | OR (95% CI) | *P-*value |
| Discovery set (n = 127) |  |  |
| Gender (female vs. male) | 0.129 (0.006–0.736) | 0.057 |
| Age (years) (≤ 60 vs. > 60) | 0.382 (0.179–0.788) | 0.010* |
| Smoking status (smoker vs. non-smoker) | 1.633 (0.691–4.030) | 0.271 |
| Histology (squamous vs. adenocarcinoma) | 2.944 (1.125–8.701) | 0.035* |
| Stage (Ib-IIb vs. IIIa) | 1.811 (0.828–4.032) | 0.139 |
| Immunotherapy (anti-PD-1 vs. anti-PD-L1) | 0.234 (0.050–0.812) | 0.034* |
| Cycles (2 vs. 3, 4) | 0.732 (0.422–1.244) | 0.254 |
| Radiological response (CR/PR vs. SD/PD) | 9.165 (4.555–21.867) | < 0.001* |
| Validation set (n = 84) |  |  |
| Gender (female vs. male) | 0.662 (0.091–3.293) | 0.635 |
| Age (years) (≤ 60 vs. > 60) | 0.352 (0.122–0.925) | 0.041* |
| Smoking status (ever vs. never) | 2.424 (0.771–9.268) | 0.153 |
| Histology (squamous vs. adenocarcinoma) | 9.750 (1.789–18.972) | 0.032* |
| Stage (Ib-IIb vs. IIIa) | 0.725 (0.278–1.822) | 0.500 |
| Immunotherapy (anti-PD-1 vs. anti-PD-L1) | 0.134 (0.019–0.604) | 0.016* |
| Cycles (2 vs. 3, 4) | 1.271 (0.723–2.333) | 0.416 |
| Radiological response (CR/PR vs. SD/PD) | 16.609 (5.086–30.559) | < 0.001* |

Abbreviations: OR, odds ratio; CI, confidence interval; CR, complete response; PR, partial response; SD, stable disease; PD, progressive disease. **P-*value < 0.05.
